# Optimization of SPME–GC–MS and characterization of floral scents from *Aquilegia japonica* and *A. amurensis* flowers

**DOI:** 10.1186/s13065-021-00754-1

**Published:** 2021-04-22

**Authors:** Hua-Ying Wang, Wei Zhang, Jian-Hua Dong, Hao Wu, Yuan-Hong Wang, Hong-Xing Xiao

**Affiliations:** 1 Key Laboratory of Molecular Epigenetics of Ministry of Education, Northeast Normal University, Changchun, 130024 China; 2 Faculty of Chemistry, Northeast Normal University, Changchun, 130024 China

**Keywords:** Columbines, VOCs, GC–MS, SPME, Northeast China

## Abstract

**Background:**

The floral scents of plants play a key role in plant reproduction through the communication between plants and pollinators. *Aquilegia* as a model species for studying evolution, however, there have been few studies on the floral scents and relationships between floral scents and pollination for *Aquilegia* taxa.

**Methods:**

In this study, three types of solid-phase micro-extraction (SPME) fiber coatings (DVB/PDMS, CAR/PDMS, DVB/CAR/PDMS) were evaluated for their performance in extracting volatile organic compounds (VOCs) from flowers of *Aquilegia amurensis*, which can contribute to the future studies of elucidating the role of floral scents in the pollination process.

**Results:**

In total, 55 VOCs were identified, and among them, 50, 47 and 45 VOCs were extracted by the DVB/CAR/PDMS fiber, CAR/PDMS fiber and DVB/PDMS fibers, respectively. Only 30 VOCs were detected in *A. japonica* taxa. Furthermore, the relative contents of 8 VOCs were significant different (VIP > 1 and *p* < 0.05) between the *A. amurensis* and *A. japonica*.

**Conclusions:**

The results can be applied in new studies of the relationships between the chemical composition of floral scents and the processes of attraction of pollinator. It may provide new ideas for rapid evolution and frequent interspecific hybridization of *Aquilegia*.

**Supplementary Information:**

The online version contains supplementary material available at 10.1186/s13065-021-00754-1.

## Background

Volatile organic compounds (VOCs), emitted by plant organs such as leaves, flowers and fruits, have served multiple biological functions, including defense against pathogens, parasites, herbivores and interactions with pollinators [1]. Among them, floral aromas are important in the reproductive processes of many plants by attracting pollinators. Traits with a large effect on pollinator preference could play an important role in the evolution of plant reproductive isolation and speciation [2–4]. In addition, it has been reported that diversification of the North American clade of *Aquilegia* (Columbines) was associated mainly with the difference in pollinators [5]. Researchers have studied the relationships between floral morphologies and pollinators. For example, the changes in nectar spur length and flower orientation are highly correlated with the shifts of pollinators from bee to hummingbird to hawkmoth [6]. Moreover, most attempts to classify interactions between insects and flowers have focused on floral odors [7]. For instance, *Mimulus lewisii* with three monoterpene volatiles can attract bumblebee pollinators, but due to the lack of the above three specific monoterpenes in its sister species *M. cardinalis*, the pollinator is not bumblebee, so the reproductive isolation between the two sister species can be maintained [4]. Similarly, a single volatile compound (indole) present in flowers of *Ipomosis tenuituba* but not its sister species *I. aggregata*, which can attract hawkmoths to flowers [8]. This information says little, however, about the relationships between floral scents and pollination, evolution, and phylogeny of *Aquilegia* taxa. Until now, approximately 1700 chemical compounds identified in floral scent have been isolated from more than 90 plant families [9]. Among these compounds, the monoterpenes limonene, (E)-β-ocimene, myrcene, linalool, α- and β-pinene, and the benzenoids benzaldehyde, methyl 2-hydroxybenzoate (methyl salicylate), benzyl alcohol, and 2-phenyl ethanol are most common [10].

In our study, using headspace solid-phase micro extraction coupled with gas chromatography–mass spectrometry (SPME–GC–MS), which is common method in the detection of VOCs, the floral scent characteristics of *Aquilegia japonica* and *A. amurensis* were evaluated. *A. japonica* populations are distributed in Northeast of China, North Korea, South Korea and Japan, while *A. amurensis* populations are restricted to the northern Greater Khingan Mountains of China, Siberia and Mongolia. *A. japonica* and *A. amurensis* are sister species, both of two species with different distribution areas are difficult to identify in nature because of their highly similar shape morphology traits. Therefore, Flora of China holds that both of two species are one species [11]. However, the analysis based on genome showed that the differentiation of the two species was obvious (unpublished). Thus, research focusing on the distribution and combination of floral scent compounds at species and subspecies levels may be of the utmost importance for understanding the molecules responsible for attracting pollinators and promoting adaptations and evolutionary processes in angiosperms.

In the analysis of the VOCs, the SPME technique is characterized by its simplicity, speed and sensitivity. It is a convenient sample preparation technique that can be followed by thermal desorption directly in an analytical instrument [12, 13]. Recently, several types of SPME fiber coatings have become available for the extraction of analytes, such as nonpolar polydimethylsiloxane (PDMS) fibers, carboxen–polydimethylsiloxane (CAR–PDMS) fibers, polydimethylsiloxane/divinylbenzene (PDMS/DVB) fibers and divinylbenzene/carboxen/polydimethyl siloxane (DVB/CAR/PDMS) fibers. Furthermore, due to the different compounds that make up the floral scents of different plant taxa, researchers use different types of fiber to study them, for example, Fan et al. [14] used PDMS/DVB fibers for *Malus* plants; Gao et al. [15] used CAR-PDMS fibers for *Freesia* × hybrid and Mohammed et al. [16] used DVB/CAR/PDMS fibers for Rose; Silva et al. [17] found that PDMS fiber in melon flowers has poor adsorption for polar compounds. In addition, previous studies have observed molecules with polarity such as protoanemonin, nonanal, dimethoxytoluene, 2-phenyl ethanol and phenyl acetaldehyde in *Aquilegia*’s floral scents [18]. Therefore, in the present study, SPME fibers coated with PDMS/DVB (65 μm), CAR/PDMS (75 μm) and DVB/CAR/PDMS (50/30 μm) were used to identify fibers suitable for measuring the floral scents in *Aquilegia*. Consequently, our study has not only assessed the performance of different fibers in extracting the VOCs of *Aquilegia* flowers, but also evaluated the main differences in compounds among the two taxa and provided fundamental information for the scent traits of *Aquilegia*.

## Methods

### Plant material

The materials were cultivated in a garden from 2017 at Changchun, Jilin, China, including eight individuals of *A. amurensis* (52.308 N, 124.376 E) and five individuals of *A. japonica* (41.949 N, 127.925 E) collected from the wild. Fully expanded flowers of the same size, were collected at around between 9 and 10 a.m. In order to reduce the difference between individuals in intraspecies, the flowers collected from different individuals of *A. amurensis* should be mixed and then approximately 0.6 g was weighted and sealed into 20 mL solid-phase micro extraction (SPME) vials (Agilent Technologies, Germany) immediately for further analysis. *A. japonica* was also done the same treated. Nine samples of *A. amurensis* were selected to set three replicates for each fiber and explore the best coating of SPME fiber. Then, six samples of *A. japonica* were collected and extracted with the best coating of fiber to discriminate different scent intensities of *Aquilegia* taxa. In addition, an admixture of a certain number of accurately weighted *n*-alkanes (C7–C30) diluted with hexane (w = 5%) was used as a standard.

### Gas chromatography–mass spectrometry experiments

To select an efficient type of fiber coating to extract volatile compounds from the flowers, SPME fibers with three different coatings were used: 65 μm DVB/PDMS (divinylbenzene/polydimethylsiloxane), 75 μm CAR/PDMS (carboxen/polydimethylsiloxane) and 50/30 μm DVB/CAR/PDMS (divinylbenzene/carboxen/polydimethylsiloxane) (Supelco, Bellefonte, PA, USA). Prior to the analyses, fibers were conditioned for 30 min according to the temperature recommended by the manufacturer. After 10 min equilibration between the flower and the headspace, the SPME fiber was exposed to the headspace of the capped vial to absorb volatile compounds of each sample under heating at 60 °C for 30 min and for 10 min at room temperature. After extraction, the fiber was removed from the flask and immediately inserted into the gas chromatograph injector (GC–MS) for 3 min for thermal desorption at 240 °C.

The flower samples were analyzed and identified using a GC–MS Agilent 7890b gas chromatograph coupled with a 5977b mass spectrometer. Chromatographic separation (GC) was performed using a DB-5MS capillary column (30 m × 0.25 mm × 0.25 μm film thickness, Agilent Technologies, Wilmington, DE, USA). The analytical conditions used were as follows: spitless injection at 240 °C; helium as the carrier gas at a flow rate of 1.0 mL/min; and GC column temperature program of GC was initially set at 40 °C for 2 min, then heated to 150 °C for 3 °C/min, maintained for 5 min, and finally increased to 250 °C at 20 °C/min and maintained for 8 min. For MS detection, an electron impact (EI) ionization system was used at 70 eV; the temperature of the transfer line and ionization source was 150 and 230 °C, respectively; and full-scan acquisition mode was performed with a mass range of 20–550 Da. Constituents were identified by comparing mass spectra with the National Institute of Standards and Technology (NIST) 14 library (similarity > 75%) and with published data (NIST, http://webbook.nist.gov/chemistry/; PubChem, http://pubchem.ncbi.nlm.nih.gov/). Moreover, the retention time of various compounds in the standard was measured according to the above experimental conditions. According to the retention time of compounds in the floral scents and n-alkanes in the standard, the retention index (RI) was calculated, and compared with the RI in the literature to further determine the components in the floral scents. In addition, relative amounts of compounds were calculated in relation to the total area of the chromatogram by normalizing the peak area (Chemstation B.07.05).

### Comparison of compound extraction sensitivity

The extraction sensitivity of various compounds was evaluated by the cumulative area normalization value (CANV) [19]. The CANV is calculated in three steps as follows:

$${\text{AVk}} = {{\left[ {{\text{Ak}}\left( {{\text{PDMS}}/{\text{DVB}}} \right) + {\text{Ak}}\left( {{\text{CAR}}/{\text{PDMS}}} \right) + {\text{Ak}}\left( {{\text{DVB}}/{\text{CAR}}/{\text{PDMS}}} \right)} \right]} \mathord{\left/ {\vphantom {{\left[ {{\text{Ak}}\left( {{\text{PDMS}}/{\text{DVB}}} \right) + {\text{Ak}}\left( {{\text{CAR}}/{\text{PDMS}}} \right) + {\text{Ak}}\left( {{\text{DVB}}/{\text{CAR}}/{\text{PDMS}}} \right)} \right]} 3}} \right. \kern-\nulldelimiterspace} 3};$$

$${\text{NAk}}\left( {\text{X}} \right) = {{{\text{Ak}}\left( {\text{X}} \right)} \mathord{\left/ {\vphantom {{{\text{Ak}}\left( {\text{X}} \right)} {{\text{AVk}}}}} \right. \kern-\nulldelimiterspace} {{\text{AVk}}}};$$

$${\text{CAk}}({\text{X}}) = \mathop \sum \limits_{n = 1}^{\infty } {\text{NAn}}\left( {\text{X}} \right).$$

In the equations: AVk is the average peak area of compound K measured by the three SPME fibers; Ak(X) is the absolute peak area of compound K extracted by the X SPME fiber, where X is any of the PDMS/DVB, CAR/PDMS and DVB/CAR/PDMS SPME fibers; NAk(X) is the standardized value of peak area of compound K extracted by the X fiber; and CAk(X) is the cumulative area normalization value of one to more compounds extracted by the X fiber. When the CANV is larger, the sensitivity of the SPME fiber is considered to be higher.

### Characterization of VOCs from *A. japonica* and *A. amurensis* flowers

One-way analysis of variance (ANOVA) using R software was performed to investigate the significant differences (p < 0.05) in the relative amounts of compounds between the two taxa. The GC–MS dataset was imported to SIMCA-P 14.1 software for statistical analysis. Principal component analysis (PCA) and partial least squares-discriminant analysis (PLS-DA) were used to differentiate the samples and identify marker metabolites. Afterwards, the variable influence on projection (VIP), which summarizes the importance of the X-variables in the PLS-DA model with many components, was used to illustrate the variables that contributed to the separation.

## Results

### Fiber performance

**Fig. 1:**
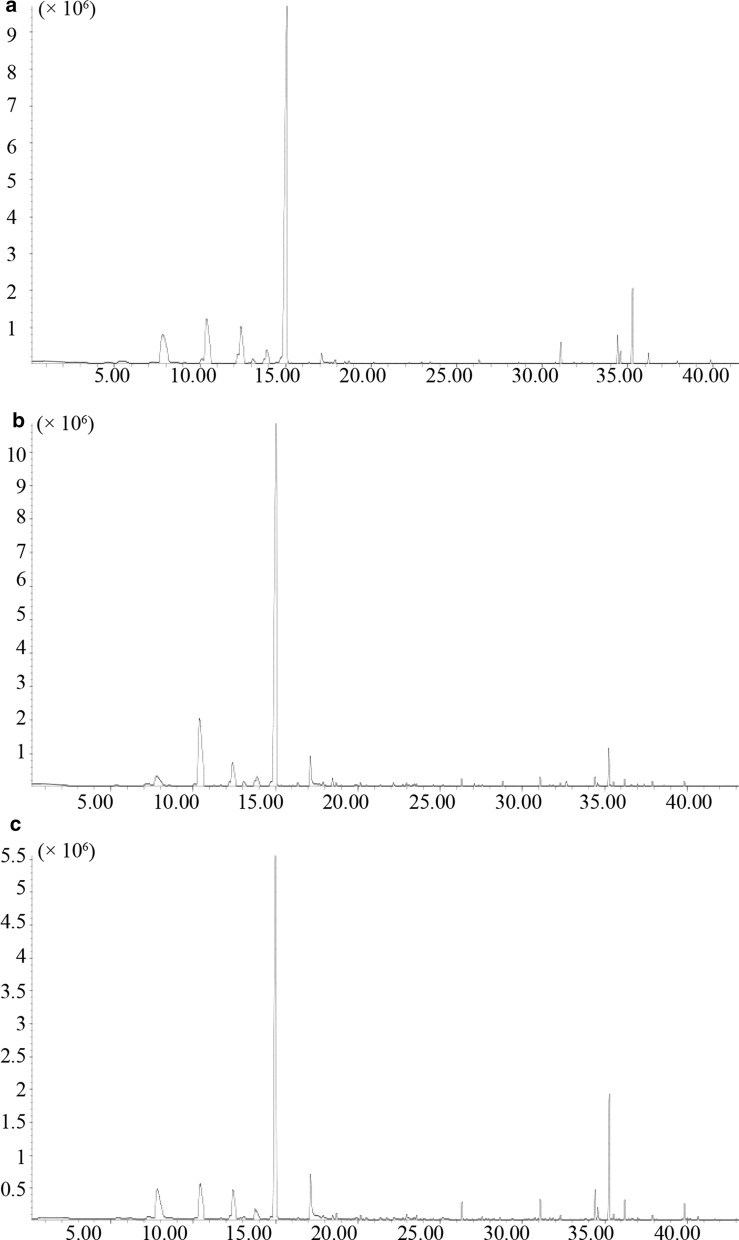
Total ion chromatogram of VOCs collected from SPME fibers coating with DVB/PDMS (**a**), CAR/PDMS (**b**) and DVB/CAR/PDMS (**c**), respectively. The x axis represents retention time (min) and the y axis represents relative abundance

Three kinds of SPME fibers were used for the SPME-GC/MS full scan analysis of *A. amurensis* samples. The total ion chromatogram was shown in Fig. 1 and clear ion spectrum was obtained. In our study, three types of fiber coatings (DVB/PDMS, CAR/PDMS, DVB/CAR/PDMS) were evaluated for their performance in absorbing VOCs, which was determined based on the number of chromatographic peaks that they detected, from flowers of columbines. In total, 55 volatile compounds were identified through MS and RI analysis, belonging to the following different chemical classes: fatty acid derivatives (10), benzenoids (2), monoterpenoids (23) and sesquiterpenoids (20) (Additional file 2: Table S1). Among them, 50 volatile compounds were extracted by DVB/CAR/PDMS fiber, 47 volatile compounds were extracted by CAR/PDMS fiber and 45 volatile compounds were extracted by the DVB/PDMS fiber. The correlation between the three repetitions of each fiber in the detection of compounds was shown in Additional file 3: Table S2. The CANV of DVB/CAR/PDMS, CAR/PDMS and DVB/PDMS fibers was 85.12, 56.16 and 29.72, respectively. Therefore, the DVB/CAR/PDMS fiber showed the best efficiency and was used to extract volatile compounds in *A. japonica*.

In addition, 39 compounds were common to the three types of fiber used, and the most abundant compounds were d-limonene (46.51%), 1R-α-pinene (10.91%), γ-muurolene (7.75%), (−)-β-pinene (7.63%) and 1-hexanol (6.39%), accounting for approximately 79% of the total GC peak area. However, a few of scarce compounds were adsorbed only by one type of fiber. Specifically, the CAR/PDMS fiber exclusively extracted 4 compounds (longifolene-(V4), α-farnesene, 1-methyl-4-(6-methylhept-5-en-2-yl)cyclohexa-1,3-diene and β-sesquiphellandrene), while 2 compounds (viridiflorene, 2-isopropenyl-5-methylhex-4-enal) were extracted only by the DVB/CAR/PDMS fiber. Additionally, m-cymene was detected only when using the DVB/PDMS fiber. Furthermore, there were 5 compounds that just the CAR/PDMS fiber did not extracted, (−)-terpinen-4-ol, verbenone, benzene, 1-methoxy-4-methyl-2-(1-methylethyl)-, myrtenyl acetate and viridiflorol. In addition, there were another 4 compounds that just the DVB/PDMS fiber did not extracted: decanal, pentanoic acid 2,2,4-trimethyl-3-carboxyisopropyl isobutyl ester, benzoic acid-ethyl ester and β-bisabolene.

### Discrimination of the different taxa

The identified compounds and their relative contents (%) in *A. japonica* flowers were analyzed using DVB/CAR/PDMS-coated SPME fiber because this type of fiber was more efficient for the extraction of compounds. In order to ensure the accuracy, 6 repetitions were set. A total of 30 volatile compounds were putatively identified in this taxon through MS and RI analysis, including fatty acid derivatives (15), benzenoids (2) and monoterpenoids (13) (Table 1). The correlation between the six replicates was shown in Additional file 4: Table S3. Furthermore, 12 analytes were not detected in *A. amurensis* taxa (15.75% of the total content in *A. japonica*), and 32 volatile compounds were not detected in *A. japonica* taxa (18.49% of the total content in *A. amurensis*).

**Table 1:** Volatile compounds identified in the flowers of two *Aquilegia* taxa extracted by the fibers DVB/CAR/PDMS

Compounds		RT	Mean Relative Content (%)		RI	RI	VIP
			*A. amurensis*	*A. japonica*	Measurements value	Reference value	
Fatty acid derivatives							
C_6_H_12_O	Hexanal	5.429	0.155	0.655	817	803	0.699**
C_6_H_10_O	3-Hexenal, (Z)-	5.548	ND	0.209	820	814	0.436
**C**_**6**_**H**_**12**_**O**	**3-Hexen-1-ol, (E)-**	**5.680**	**ND**	**4.604**	**824**	**842**	1.981*
C_6_H_12_O	Cyclobutanol, 2-ethyl-	5.796	0.122	ND	827	828	0.316**
C_6_H_14_O	1-Hexanol	6.435	6.393	9.193	843	838	1.757
C_7_H_14_O	Heptanal	8.010	ND	0.081	883	899	0.180
C_8_H_16_O	Octanal	13.220	0.909	1.622	996	1005	0.928
**C**_**8**_**H**_**14**_**O**_**2**_	**3-Hexen-1-ol, acetate, (Z)-**	**13.340**	**ND**	**4.929**	**998**	**1025**	2.052*
C_8_H_18_O	1-Octanol	16.931	3.237	4.007	1071	1069	0.885
C_11_H_24_	Undecane	18.320	ND	0.349	1098	1100	0.518
C_9_H_18_O	1-Nonanal	18.573	0.394	0.468	1104	1105	0.344
C_9_H_18_O_2_	Octanoic acid, methyl ester	19.450	ND	0.130	1122	1128	0.329*
C_10_H_20_O	Decanal	23.570	0.255	0.348	1207	1208	0.311
**C**_**11**_**H**_**22**_**O**_**2**_	**Methyl decanoate**	**28.910**	**ND**	**1.935**	**1323**	**1325**	1.328**
C_14_H_20_	Bicyclo[4.1.0]heptane, 7-bicyclo[4.1.0]hept-7-ylidene-	31.929	0.167	ND	1392	1427	0.206
C_16_H_30_O_4_	Pentanoic acid, 2,2,4-trimethyl-3-carboxyisopropyl, isobutyl ester	39.840	0.073	ND	1584	1581	0.149
C_16_H_30_O_4_	2,2,4-Trimethyl-1,3-pentanediol diisobutyrate	39.939	0.506	1.214	1586	1588	0.788**
C_17_H_34_O_2_	Methyl palmitate	48.070	ND	0.739	1929	1905	0.822**
Benzenoids							
C_7_H_6_O	Benzaldehyde	10.870	ND	0.339	946	954	0.454
C_9_H_10_O_2_	Benzoic acid, ethyl ester	21.470	0.262	ND	1163	1170	0.256
C_8_H_8_O_3_	Methyl salicylate	22.731	0.137	0.180	1189	1190	0.185
Monoterpenoids							
C_10_H_16_	α-Thujene	9.176	0.207	ND	911	931	0.435**
**C**_**10**_**H**_**16**_	**1R-α-Pinene**	**9.495**	**10.911**	**2.323**	**918**	**922**	2.777**
C_10_H_16_	Bicyclo[3.1.1]heptane, 6,6-dimethyl-2-methylene-, (1*S*)-	11.560	0.500	0.913	961	978.6	0.591**
**C**_**10**_**H**_**16**_	**(**−**)-β-Pinene**	**11.765**	**7.628**	**0.719**	**965**	**979**	2.510**
C_10_H_16_	β-Myrcene	12.470	0.972	2.677	980	991	1.377
**C**_**10**_**H**_**16**_	**3-Carene**	**13.431**	**1.287**	**ND**	**1000**	**1021**	1.016*
C_10_H_14_	Cycloheptane, 1,3,5-tris(methylene)-	14.160	ND	0.804	1015	1039	0.620
C_10_H_14_	*O*-Cymene	14.330	0.371	0.180	1019	1006	0.508*
C_10_H_16_	d-Limonene	14.650	46.505	55.709	1025	1033	3.164
C_10_H_16_	*trans*-Ocimene	15.350	0.030	ND	1039	1049	0.128*
C_10_H_16_	*cis*-β-Ocimene	15.591	0.124	ND	1044	1038	0.327**
C_10_H_16_	γ-Terpinene	16.074	0.312	0.125	1053	1061	0.465**
C_10_H_16_	Terpinolen	17.462	0.223	ND	1081	1087	0.453**
C_10_H_18_O	Linalool	18.220	0.482	0.036	1096	1098	0.626**
C_10_H_14_	*p*-Mentha-1,5,8-triene	18.730	ND	0.306	1107	1097	0.316
C_10_H_16_	(E,Z)-2,6-Dimethylocta-2,4,6-triene	19.840	0.151	ND	1130	1129	0.288*
C_10_H_16_O	(+)-(E)-Limonene oxide	20.160	0.193	0.341	1136	1146	0.385
C_10_H_16_O	2-Isopropenyl-5-methylhex-4-enal	22.164	0.201	ND	1178	1198	0.340
C_10_H_18_O	(−)-Terpinen-4-ol	22.236	0.111	ND	1179	1175	0.183
C_10_H_18_O	α-Terpineol	22.960	0.261	ND	1194	1194	0.491**
C_10_H_16_O	2-Cyclohexen-1-ol,2-methyl-5-(1-methylethenyl)-,cis	23.080	ND	4.183	1196	1207	1.173
C_10_H_14_O	Verbenone	23.450	0.169	ND	1204	1204	0.226
C_11_H_16_O	Thymol methyl ether	24.620	0.075	ND	1229	1162	0.254**
C_10_H_14_O	2-Cyclohexene-1-one,3-Methyl-6-(1-methylethenyl)-, (*S*)-	26.347	0.455	0.687	1267	1279	0.350
C_12_H_18_O_2_	Myrtenyl acetate	28.682	0.220	ND	1320	1306	0.440**
Sesquiterpenoids							
C_15_H_24_	1*H*-Cycloprop[e]azulene, decahydro-1,1,7-trimethyl-4-methylene-	30.831	0.164	ND	1367	1386	0.204
C_15_H_24_	Copaene	31.163	1.061	ND	1375	1388	0.967**
C_15_H_24_	Zingiberene	31.736	0.146	ND	1388	1412	0.271*
C_15_H_24_	1*H*-Cycloprop[e]azulene, 1a,2,3,4,4a,5,6,7b-octahydro-1,1,4,7-tetramethyl-, [1a*R*-(1aα,4α,4aβ,7bα)]-	32.388	0.367	ND	1403	1419	0.429*
C_15_H_24_	Caryophyllene	32.992	0.060	ND	1417	1424	0.123
C_15_H_24_	1,5,9,9-Tetramethyl-1,4,7-cycloundecatriene	34.494	1.436	ND	1454	1476	1.038
C15H24	(−)-Alloaromadendrene	34.669	0.750	ND	1458	1435	0.816**
**C**_**15**_**H**_**24**_	**γ-Muurolene**	**35.394**	**7.751**	**ND**	**1475**	**1475**	2.636**
C_15_H_24_	α-Curcumene	35.629	0.488	ND	1481	1483	0.495*
C_15_H_24_	Viridiflorene	35.979	0.104	ND	1490	1484	0.247*
**C**_**15**_**H**_**24**_	**α-Muurolene**	**36.311**	**1.354**	**ND**	**1498**	**1501**	1.103**
C_15_H_24_	β-Bisabolene	36.727	0.210	ND	1507	1506	0.327*
C_15_H_24_	Cadina-1(10),4-diene	37.096	0.234	ND	1517	1531	0.370*
C_15_H_24_O	α-Copaen-11-ol	37.989	0.609	ND	1538	1537	0.595*
C_15_H_26_O	Viridiflorol	40.107	0.218	ND	1589	1580	0.336
C_15_H_26_O	α-Bisabolol	44.628	1.046	ND	1690	1680	0.728*

VOCs with significant differences are shown in bold

*RT* retention time, *ND* not detected, *RI* retention index, *VIP* variable importance in projection

*Represents significant differences between different taxa 0.01 < p < 0.05

**Represents significant differences between different taxa p < 0.01

In addition, the main floral scents in *A. amurensis* were d-limonene and 1*R*-α-pinene (46.51% and 10.91% of the total content, respectively) while the primary volatile components in *A. japonica* included d-limonene and 1-hexanol (constituting 55.72% and 9.19% of the total, respectively) (Table 1). The relative contents of the different chemical classes (fatty acid derivatives, benzenoids, monoterpenoids and sesquiterpenoids) between the two taxa were calculated and compared (Additional file 1: Figure S1). The kinds of terpenes were more in *A. amurensis* than in *A. japonica*, and sesquiterpenoids were not detected in *A. japonica*. However, the kinds of fatty acid derivatives in *A. japonica* was more than that in another taxon (Additional file 1: Figure S1).

**Fig. 2:**
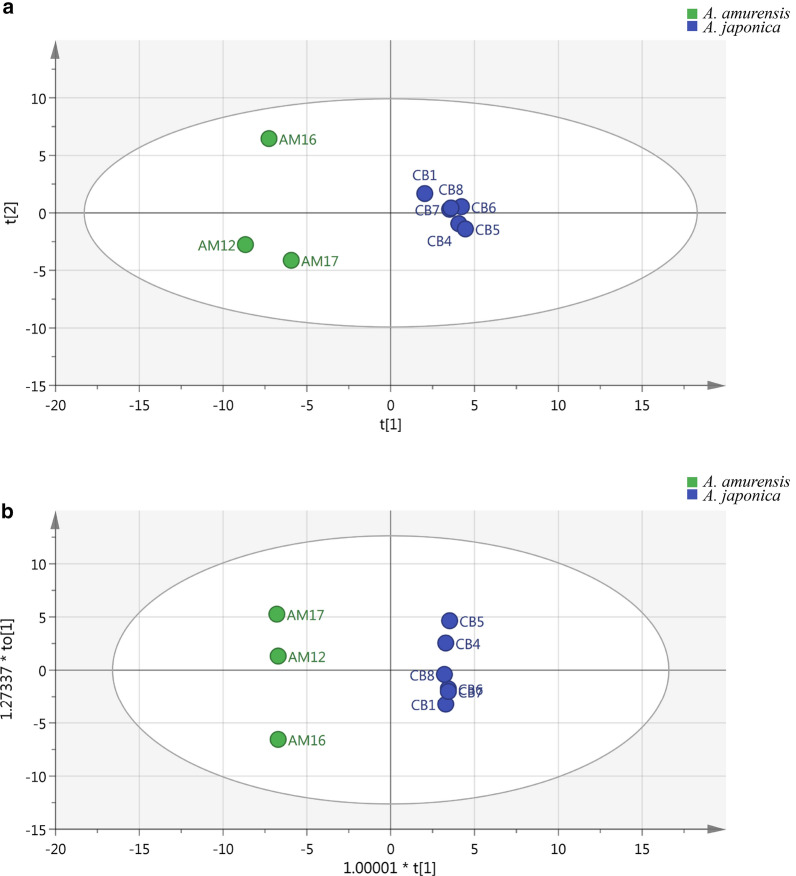
The PCA score plots (**a**) and PLS-DA score plots (**b**) for datasets of GC–MS from the two taxa

Moreover, PCA, an unbiased statistical approach, was used to evaluate the separation of the different taxa (Fig. 2a). The two taxa were clearly separated and located in the positive and negative axes of PC1. However, the model described 48.5% of the variation (R2X (cum) = 0.918). Then, a supervised method, PLS-DA, was applied, and the PLS-DA score plot showed a good separation (R2X(cum) = 0.852, R2Y(cum) = 1, Q2(cum) = 0.952) (Fig. 2b). Furthermore, variables with VIP > 1 were considered important for the discrimination of samples in the PLS-DA score plot. This result indicated that the compounds (E)-3-hexen-1-ol, (Z)-3-hexen-1-ol acetate, methyl decanoate, 1R-α-pinene, (−)-β-pinene, 3-carene, γ-murolene and α-muurolene compounds were probably responsible for the observed separation (VIP > 1, p < 0.05) (Table 1), constituting 28.93% and 14.51% of the total content in *A. amurensis* and *A. japonica*, respectively.

## Discussion

### Fiber selection

The choice of the most appropriate fiber is made to cover as many metabolites as possible. To select the most efficient fiber coating for the extraction of VOCs in the *Aquilegia* taxa, three SPME fibers were used. In our study, the DVB/CAR/PDMS fiber exhibited better extraction efficiency than the DVB/PDMS and CAR/PDMS fibers, presenting the highest CANV (85.12) compared to the other fibers (56.16 and 29.72, respectively). The affinity of the fiber for an analyte depends on the principle of ‘like dissolves like’. Previous studies have demonstrated that many polar molecules in the *Aquilegia*’s flora scents [18] as well as the DVB/CAR/PDMS fiber had an intermediate polarity and some studies also confirmed its efficiency [20, 21]. The high efficiency may be because the coating with three different components improves the ability to adsorb compounds [22]. The DVB/PDMS fiber is preferred for the extraction of analytes with higher molecular weights (MW 50–300), such as volatiles, amines, and nitroaromatic compounds. Specifically, 8 fatty acid derivatives, 1 benzenoids and 36 terpenes were identified using the DVB/PDMS fiber, fewer than those detected by the other two fiber types. However, the CAR/PDMS fiber is more efficient for the extraction of gasses and low molecular weight compounds (MW 30–225) [23]. Among the 5 compounds that just the CAR/PDMS fiber did not extracted, most have intermediate and higher molecular weights, which was consistent with the results of the Silva et al. [17].

Moreover, these adsorbent type coatings, carried out by sorption of analytes in internal pores, are formed by porous solids. Therefore, saturation of the surface available for adsorption occurs because of the limited thickness. Competition between compounds was more intense when used the CAR/PDMS fiber than used the DVB/CAR/PDMS. When considering repeatability, the CAR/PDMS fiber was better than the 50/30 m DVB/CAR/PDMS fiber, and Kataoka et al. also reported this result [24]. However, when considering sensitivity, the DVB/CAR/PDMS fiber showed higher performance than that of CAR/PDMS and DVB/PDMS. Thus, the DVB/CAR/PDMS fiber has been selected for use in the measurement of the floral scents of *Aquilegia* and six replicate *A. japonica* flowers were evaluated when using the DVB/CAR/PDMS fiber.

### Scent composition in relation to the pollinators of the two *Aquilegia* taxa

Speciation in radiating flowering plants is often accompanied by diversification of animal pollinators [24–26]. Perhaps the most well-known signal in *Aquilegia* is floral color, orientation and the structure of spurs [6, 27]. Meanwhile, the roles of floral scents have been investigated in other systems [28–30], showing that the floral scents are important signals for communication between plants and pollinators, representing an important cue for pollinators [31, 32]. Therefore, a prezygotic reproductive barrier is expected when the composition of the floral scent is different. For example, Huber et al. [33] proposed that flowers of two *Gymnadenia* species with different floral odors, as well as other floral traits such as color and spur length, attracted different pollinators, enhancing prezygotic isolation.

The variability of floral scents among entomophilous plants has been reported to depend on the reliance on different pollinator groups with different olfactory preferences [34]. For example, the high relative content of the most volatile monoterpene alkenes (e.g. limonene) in the floral scent of *Silene gallica* and *S. coeli-rosa* pollinated by bees has suggested that these compounds are used as attractants of bees [35]. Jürgens and Dötterl investigated floral scents of four *Aquilegia* taxa, *A. vulgaris*, *A. canadensis*, *A. chrysantha* and *A. glandulosa* [18]. They found that the dominant compound of these four *Aquilegia* species was octanal (29.5–42%). In contrast, high relative amounts of the monoterpene d-limonene, 46.51% for *A. amurensis* and 55.71% for *A. japonica* were detected. The individuals were selected for the experiment that produced much less octanal, 0.91% and 1.62% for *A. amurensis* and *A. japonica*, respectively. There may be two reasons for this difference: one is that Jürgens et al. did not use SPME to detect the VOCs *Aquilegia*. Different detection methods lead to different compounds of the floral scent compounds of *Aquilegia* in different regions. In future research, we should increase the species of samples and use the same method to measure the VOCs of *Aquilegia*; the other reason is that they are located in order to adapt to different pollinators, *Aquilegia* in different regions have different VOCs.

Our study has identified that the floral scents of the two taxa are dominated by the same one compound (d-limonene), suggesting an adaptation to the same pollinator. Nevertheless, the low-abundance scent components may be effective specific attractants of potential pollinators and cannot be ignored [29]. For instance, the main floral scent compound of the floral four *Aquilegia* species that Jürgens and Dötterl studied was octanal (29.5–42%), but the pollinators for these species were varied. The visitation of *A. chrysantha* was visited by hawk moths may correlate with relatively high amount of 2-phenyl ethanol (13.5%) compared to that of the other three *Aquilegia* species [29]. Therefore, the fact that the two taxa share the same main floral scent components may be attributed to their closer phylogenetic relationship.

The notable differences between the taxa were the increase in the relative amounts of fatty acid derivatives and the decrease in the relative amounts of monoterpenoids in *A. japonica* and the detection of various sesquiterpenes only in *A. amurensis*. Among the fatty acid derivatives, the relative proportions of (Z)-3-hexen-1-ol acetate, (E)-3-hexen-1-ol and methyl decanoate (VIP > 1, *p* < 0.05) were significantly different between the two species, representing nearly 10% of the total floral scents of *A. japonica* but not detected in *A. amurensis*. However, (Z)-3-hexen-1-ol acetate is often released from vegetation rapidly after damage [36]. It can be hypothesized that this compound may have a defense function. The large number of low-abundance sesquiterpenoids in *A. amurensis* may represent biosynthetic byproducts, as the monoterpenes and sesquiterpenes are derived from the mevalonic acid pathway via farnesyl pyrophosphate [37]. Further experiments are necessary to draw conclusions regarding whether these sesquiterpenes are byproducts or serve critical functions in plant pollinator relationships, further experiments are necessary to draw conclusions.

## Conclusions

In this study, by evaluating the properties of different coatings of SPME fibers, the method of extracting and identifying the VOCs of *Aquilegia* flowers can be optimized. The DVB/CAR/PDMS fiber had the good performance, including sensitivity and repeatability, which is suitable for the subsequent detection of *Aquilegia* floral scent compounds. In the flowers of two sister species of *A. japonica* and *A. amurensis*, except for the main component of VOCs was d-limonene, there were significant differences in the types and relative content of fatty acid derivatives and terpenoid. The types and relative content of fatty acid derivatives in *A. japonica* were higher than those of *A. amurensis*, while the types and relative content of monoterpenes were lower than those of *A. amurensis*, and no sesquiterpenes were detected in *A. japonica*; there were also significant differences in the contents of eight compounds, including 3-hexen-1-ol, (E)-,3-hexen-1-ol, acetate, (Z)-,methyl decanoate, 1*R*-α-pinene, (−)-β-pinene, 3-carene, γ-muurolene and α-muurolene. The result provides important information for the future studies involving the VOCs of *Aquilegia* flowers and can be applied to the new study of relationship between the chemical components of floral scents and the attraction process of pollinators.

## Supplementary Information

**Additional file 1: Figure S1.** Statistical analysis of the volatile compounds present in the flowers of the two *Aquilegia* taxa. The x axis represents the type of VOCs and the y axis represents quantity of each VOCs.

**Additional file 2: Table S1.** Comparison of three SPME fibers for the extraction of volatile compounds identified in the flowers of *A. amurensis*. The character 0, 1, 2, 3 represents the number of times the VOCs have been detected.

**Additional file 3: Table S2.** The correlation between the three repetitions of each fiber in the detection of compounds. (a) DVB/CAR/PDMS; (b) PDMS/DVB; (c) CAR/PDMS.

**Additional file 4: Table S3.** The correlation between the six replicates in *A. japonica*.

**Additional file 5: Table S4.** All data generated or analyzed during this study.

## Data Availability

All data generated or analyzed during this study are included in this published article and its Additional file 5: Table S4.

## Abbreviations

ANOVA: Analysis of variance
CANV: Cumulative area normalization value
CAR: Carboxen
DVB: Divinylbenzene
EI: Electron impact
ND: Not detected
NIST: National Institute of Standards and Technology
PCA: Principal component analysis
PDMS: Polydimethyl siloxane
PLS-DA: Partial least squares-discriminant analysis
RI: Retention index
RT: Retention time
SPME–GC–MS: Solid-phase micro extraction coupled with gas chromatography–mass spectrometry
VIP: Variable importance in projection
VOCs: Volatile organic compounds

## References

[1] Holopainen JK, Gershenzon J (2010). Multiple stress factors and the emission of plant VOCs. Trends Plant Sci.

[2] Bradshaw H, Otto KG, Frewen BE, McKay JK, Schemske DW (1998). Quantitative trait loci affecting differences in floral morphology between two species of monkeyflower (*Mimulus*). Genetics.

[3] Schemske DW, Bradshaw H (1999). Pollinator preference and the evolution of floral traits in monkeyflowers (*Mimulus*). Proc Natl Acad Sci.

[4] Bradshaw H, Schemske DW (2003). Allele substitution at a flower colour locus produces a pollinator shift in monkeyflowers. Nature.

[5] Hodges SA, Derieg NJ (2009). Adaptive radiations: from field to genomic studies. Proc Natl Acad Sci.

[6] Whittall JB, Hodges SA (2007). Pollinator shifts drive increasingly long nectar spurs in columbine flowers. Nature.

[7] Cordeiro G, Pinheiro M, Dötterl S, Alves-dos-Santos I (2017). Pollination of *Campomanesia phaea* (Myrtaceae) by night-active bees: a new nocturnal pollination system mediated by floral scent. Plant Biol.

[8] Bischoff M, Raguso RA, Jürgens A, Campbell DR (2015). Context-dependent reproductive isolation mediated by floral scent and color. Evolution.

[9] Knudsen JT, Eriksson R, Gershenzon J, Ståhl B (2006). Diversity and distribution of floral scent. Bot Rev.

[10] Dunkel M, Schmidt U, Struck S, Berger L, Gruening B, Hossbach J, Jaeger IS, Effmert U, Piechulla B, Eriksson R (2008). SuperScent—a database of flavors and scents. Nucleic Acids Res.

[11] Yang Z, Nielsen R, Hasegawa M (1998). Models of amino acid substitution and applications to mitochondrial protein evolution. Mol Biol Evol.

[12] Zhu F, Xu J, Ke Y, Huang S, Zeng F, Luan T, Ouyang G (2013). Applications of in vivo and in vitro solid-phase microextraction techniques in plant analysis: a review. Anal Chim Acta.

[13] Miguel MG (2010). Antioxidant and anti-inflammatory activities of essential oils: a short review. Molecules.

[14] Fan J, Zhang W, Zhou T, Zhang D, Zhang D, Zhang L, Wang G, Cao F (2018). Discrimination of *Malus* taxa with different scent intensities using electronic nose and gas chromatography–mass spectrometry. Sensors.

[15] Gao F, Liu B, Li M, Gao X, Fang Q, Liu C, Ding H, Wang L, Gao X (2018). Identification and characterization of terpene synthase genes accounting for volatile terpene emissions in flowers of *Freesia* × hybrida. J Exp Bot.

[16] Ibrahim M, Agarwal M, Yang JO, Abdulhussein M, Du X, Hardy G, Ren Y (2019). Plant growth regulators improve the production of volatile organic compounds in two rose varieties. Plants.

[17] Silva FAN, da Silva AA, de Sousa Fernandes N, Rodrigues THS, Canuto KM, do Nascimento RF, de Brito ES, de Aragão FAS, Freitas BM, Zocolo GJ (2018). Evaluation of headspace solid-phase microextraction gas chromatography–mass spectrometry for the characterization of volatile organic compounds from Melon (*Cucumis melo* L.) flowers. Chromatographia.

[18] Jürgens A, Dötterl S (2004). Chemical composition of anther volatiles in Ranunculaceae: genera-specific profiles in *Anemone*, *Aquilegia*, *Caltha*, *Pulsatilla*, *Ranunculus*, and *Trollius* species. Am J Bot.

[19] Tat L, Comuzzo P, Stolfo I, Battistutta F (2005). Optimization of wine headspace analysis by solid-phase microextraction capillary gas chromatography with mass spectrometric and flame ionization detection. Food Chem.

[20] Rocha SM, Caldeira M, Carrola J, Santos M, Cruz N, Duarte IF (2012). Exploring the human urine metabolomic potentialities by comprehensive two-dimensional gas chromatography coupled to time of flight mass spectrometry. J Chromatogr A.

[21] Araújo AM, Moreira N, Lima AR, de Lourdes BM, Carvalho F, Carvalho M, de Pinho PG (2018). Analysis of extracellular metabolome by HS-SPME/GC–MS: optimization and application in a pilot study to evaluate galactosamine-induced hepatotoxicity. Toxicol Lett.

[22] Zhang M, Pan Q, Yan G, Duan C (2011). Using headspace solid phase micro-extraction for analysis of aromatic compounds during alcoholic fermentation of red wine. Food Chem.

[23] Risticevic S, Lord H, Gorecki T, Arthur CL, Pawliszyn J (2010). Protocol for solid-phase microextraction method development. Nat Protoc.

[24] Kataoka H, Lord HL, Pawliszyn J (2000). Applications of solid-phase microextraction in food analysis. J Chromatogr A.

[25] Grant V (1949). Pollination systems as isolating mechanisms in angiosperms. Evolution.

[26] Stebbins GL (1970). Adaptive radiation of reproductive characteristics in angiosperms, I: pollination mechanisms. Annu Rev Ecol Syst.

[27] Miller RB, Willard CL (1983). The pollination ecology of *Aquilegia micrantha* (Ranunculaceae) in Colorado. Southwest Nat.

[28] Harder LD, Johnson SD (2009). Darwin’s beautiful contrivances: evolutionary and functional evidence for floral adaptation. New Phytol.

[29] Schiestl FP (2005). On the success of a swindle: pollination by deception in orchids. Naturwissenschaften.

[30] Yuan Y-W, Byers KJ, Bradshaw H (2013). The genetic control of flower–pollinator specificity. Curr Opin Plant Biol.

[31] Knudsen JT (2002). Variation in floral scent composition within and between populations of *Geonoma macrostachys* (Arecaceae) in the western Amazon. Am J Bot.

[32] Plepys D, Ibarra F, Löfstedt C (2002). Volatiles from flowers of *Platanthera bifolia* (Orchidaceae) attractive to the silver Y moth, *Autographa gamma* (Lepidoptera: Noctuidae). Oikos.

[33] Huber FK, Kaiser R, Sauter W, Schiestl FP (2005). Floral scent emission and pollinator attraction in two species of *Gymnadenia* (Orchidaceae). Oecologia.

[34] Dobson HE (2006). Relationship between floral fragrance composition and type of pollinator. Biology of floral scent.

[35] Jürgens A (2004). Flower scent composition in diurnal *Silene* species (Caryophyllaceae): phylogenetic constraints or adaption to flower visitors?. Biochem Syst Ecol.

[36] Kessler A, Baldwin IT (2001). Defensive function of herbivore-induced plant volatile emissions in nature. Science.

[37] Kaiser R, Müller P, Lamparsky D (1991). Perfumes: art, science and technology.

