# Supplementary figures and images for: Optimization of SPME–GC–MS and characterization of floral scents from Aquilegia japonica and A. amurensis flowers
Source: BMC Chem. 2021 Apr 22;15(1):26. doi: 10.1186/s13065-021-00754-1 (PMC8063332; doi:10.1186/s13065-021-00754-1)

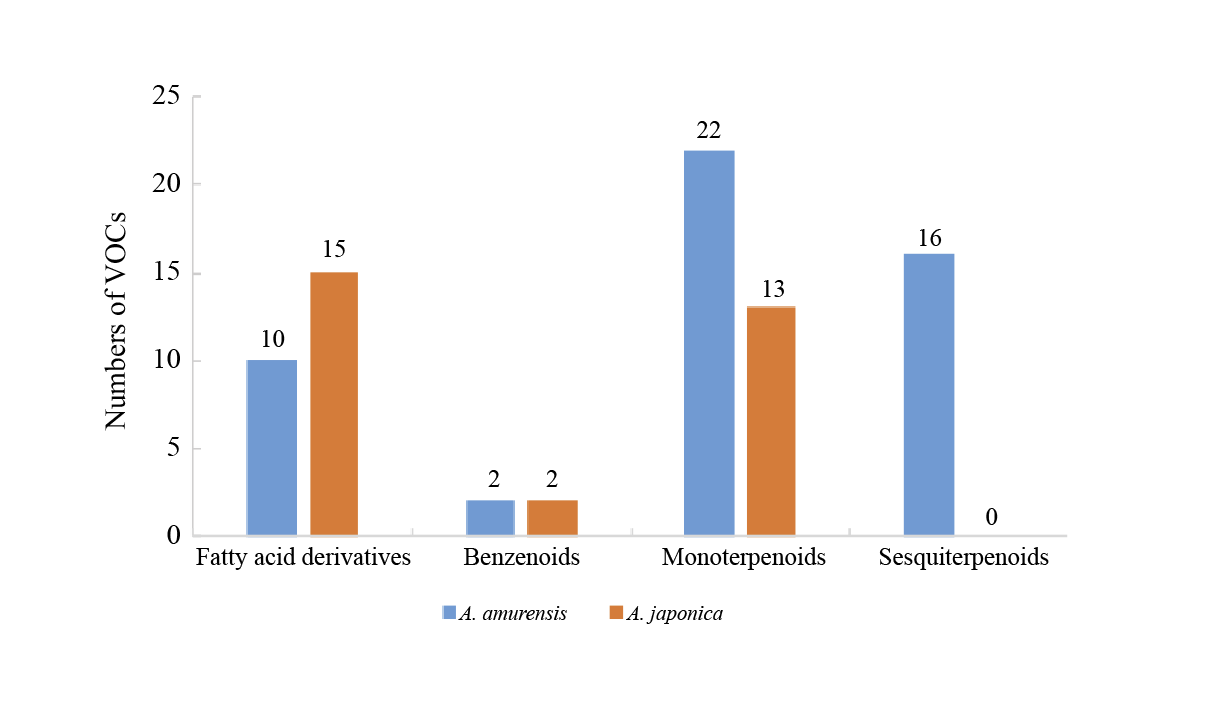

Supplement: Supplementary file 1 — Additional file 1: Figure S1. Statistical analysis of the volatile compounds present in the flowers of the two Aquilegia taxa. The x axis represents the type of VOCs and the y axis represents quantity of each VOCs. [file 13065_2021_754_MOESM1_ESM.png]
